# The demographic characteristics and prognosis of acute myeloid leukemia with t(3;3)/inv(3) in the united states: a SEER-based study

**DOI:** 10.1038/s41598-025-95783-4

**Published:** 2025-04-18

**Authors:** Xianfeng Ouyang, Xia Wu, Su Hu, Wei Jiang, Ying Guo, Yan Huang

**Affiliations:** 1 Department of Hematology, Jiujiang First People’s Hospital, Jiujiang City, 332000 Jiangxi Province China; 2 Department of Hematology, West China Hospital, Sichuan University, Chengdu City, 610041 Sichuan Province China; 3 No.48 South Taling Road, Xunyang District, Jiujiang City, Jiangxi Province China; 4 No.37 Guoxue Lane, Wuhou District, Chengdu City, Sichuan Province China

**Keywords:** Acute myeloid leukemia, Acute myeloid leukemia with t(3; 3)/inv(3), Overall survival, Cancer-specific survival, *GATA2*, *MECOM*, Cancer, Genetics

## Abstract

Acute myeloid leukemia (AML) with t(3;3) (q21; q26.2) or inv(3) (q21; q26.2) is a rare subtype of acute myeloid leukemia with a poor prognosis. Research on the demographic characteristics and clinical outcomes of AML patients with t(3;3) (q21; q26.2) or inv(3) (q21; q26.2) is limited. All AML patients diagnosed with t(3;3) (q21; q26.2) or inv(3) (q21; q26.2) between 2000 and 2021 from SEER (Surveillance, Epidemiology, and End Results Program) database were included in this study. Survival analysis was performed using the Kaplan–Meier method. The log-rank test was used to analyze the significance of differences between overall survival (OS) and cancer-specific survival (CSS). Due to being a retrospective study, the limited amount of baseline characteristics available, and the missing information on treatments and recurrence, the provided dataset is not exactly representative for the US population. The median age of the total 90 AML patients with t(3;3) (q21; q26.2) or inv(3) (q21; q26.2) was 65(49–74) years old. Among them, 57.78% were married and non-Hispanic whites accounted for 74.44%. 84.44% of the patients received chemotherapy. The median OS was 8 months, and the median CSS was 10 months. The 5-year survival rate for OS was 15.29% (95%CI:7.98-24.78%), and 20.05% (95%CI:10.69–31.51%) for CSS. The OS and CSS of patients above 60 years were inferior to those of patients under 60 years. Single (never-married) patients had better OS and CSS than those with other marital status. Age and marital status at diagnosis were independent influencing factors for OS and CSS based on the multivariate COX regression analysis. AML with t(3;3) (q21; q26.2) or inv(3) (q21; q26.2) mostly occurs in adults and has poor clinical outcomes with worse OS and CSS, especially in the elderly.

**Supplementary Information:**

The online version contains supplementary material available at 10.1038/s41598-025-95783-4.

## Introduction

Acute myeloid leukemia (AML) is the most common type of acute leukemia in adults, particularly the elderly, with a median age of 68 years^1^. AML is characterized by uncontrolled proliferation, impaired differentiation and blocked apoptosis of myeloid progenitor cells, leading to infiltration of various tissues and organs, such as the bone marrow, peripheral blood, liver, spleen and lymph glands, as well as inhibition of normal hematopoietic function^2^. The standard treatment for AML has remained unchanged for decades^3^. The 5-year overall survival (OS) rate is only 30%, and even only 17% in patients over 60 years^4^. Cytogenetic abnormalities are closely associated with the prognosis of AML patients, as well as clinical treatment decisions^5^.

According to the World Health Organization (WHO) Classification, AML with t(3;3) (q21; q26.2) or inv(3) (q21; q26.2), hereinafter referred to as t(3;3)/inv(3), is a type of acute myeloid leukemia with recurrent genetic abnormalities. Both of these rearrangements, recurrent translocations and inversions of chromosome 3 in AML, lead to the translocation of the 3q21 to 3q26, namely the *GATA2* gene translocation to the *MECOM* gene^6,7^. The chromosomal rearrangement on 3q26 which is related to overexpression of the *EVI1* (also known as MECOM) gene was first reported in 1992^8^. However, the enhancer on 3q21 that results in EVI1 overexpression has not been identified for more than 20 years. The *GATA2* gene on 3q21 was identified as an enhancer that led to the overexpression of *EVI1* gene until 2014. Chromosomal rearrangements between 3q21 and 3q26 cause *EVI1* overexpression and *GATA2* haploinsufficiency^9–11^. Increased expression of *MECOM* gene has been reported in some cancers and leukemia^12–15^. In hematopoietic malignancies, approximately 10% of AML patients exhibit elevated *EVI1* gene expression, which contributes to poor survival^16^. Chromosome rearrangements between 3q21 and 3q26 occur in approximately 1–2% of all AML cases and in 10-20% of AML cases with high *EVI1* expression^17,18^. In AML with t(3;3)/inv(3), the translocated *GATA2* enhancer induces the overexpression *of EVI1* merely, although *MECOM* can be transcribed from two disparate promoters to encode two transcript isoforms *MDS1*-*EVI1* and *EVI1*. Because of the translocation of the *GATA2* oncogenic enhancer in t(3;3)/inv(3) AML, *EVI1* was overexpressed and simultaneously *GATA2* expression was suppressed from the rearranged allele^9,10^.

Based on the 2022 European leukemia network (ELN) risk stratification by genetics at AML diagnosis, t(3;3)/inv(3) is one of adverse prognostic factors^19^. AML with t(3;3)/inv(3) is a rare subtype of leukemia with relatively low incidence^5,20^. Platelet counts of t(3;3)/inv(3) AML patients are generally normal or elevated, but are not reduced too frequently. Multilineage dysplasia of bone marrow cells, especially megakaryocyte dysplasia, is very common. Chromosome 7 abnormalities and complex karyotypes are common cytogenetic abnormalities^21,22^. AML with t(3;3)/inv(3) has been identified as a distinctive subgroup with an unfavorable prognosis^23,24^. However, the knowledge of the clinical characteristics and outcomes of AML with t(3;3)/inv(3) is limited. In particular, there have been few studies on cancer-specific survival (CSS).

Till now, there are few researches about the influence of socio-demographic characteristics on prevalence and outcome of AML. Doddi et al.^25^ explored demographic disparities in AML mortality trends from 1999 to 2020 in the United States. They found that the males had a significant average Annual Percent Change (AAPC) with an overall uptrend and the White population had the greatest mortality rate.

In this study, we collected data from the SEER database to understand the current status of AML with t(3;3)/inv(3) in the United States and provide insights into demographic characteristics and survival outcomes.

## Methods and materials

### Data source

The data were obtained from the National Cancer Institute Surveillance Epidemiology and End Results (SEER) database, from 17 Population-Based Registries between 2000 and 2021 (November 2023 Submission)^26^. Cancer incidence and survival data from population-based cancer registries, collected and published by SEER (seer.cancer.gov), currently cover approximately 48.0% of the U.S. population.

## Study population

The data were collected using the SEER*Stat v8.4.0.3 software package. Our analysis was limited to code 9869/3: acute myeloid leukemia with inv(3)(q21;q26.2) or t(3;3)(q21;q26.2); RPN1-EVI1, defined in the third edition of the International Classification of Diseases for Oncology (ICD-O-3). From 2000 to 2021, AML patients with t(3;3)/inv(3) were first diagnosed in 2010. Therefore all patients of AML with t(3;3)/inv(3) diagnosed between 2010 and 2021 were included in this study.

## Study variables

The variables about demographic characteristics of AML patients with t(3;3)/inv(3) gathered from the SEER database included age at diagnosis, sex, year of diagnosis, marital status at diagnosis, race and ethnicity, median household income, chemotherapy, PRCDA (the purchased/referred care delivery areas), Rural-Urban Continuum Code, survival status, and survival time. Age at diagnosis was divided into < 60 years and ≥ 60 years for the subgroup analysis. The patients were split into two groups (2010–2015 and 2016–2021) based on the year of diagnosis. The variables of ethnicity included Hispanics and non-Hispanics, and the race contained White race, Black race, Asian or Pacific Islander and American Indian/Alaska Native. The patients were divided into two groups (<$75,000 and ≥$75,000) based on median household income. The variable of chemotherapy contained Yes and No/Unknown, and the PRCDA variable included Yes and Not. The Rural-Urban Continuum Code variable included counties in metropolitan areas with a population of 1 million, counties in metropolitan areas with a population of 250,000 to 1 million, counties in metropolitan areas with a population less than 250 thousand, nonmetropolitan counties adjacent to a metropolitan area, and nonmetropolitan counties not adjacent to a metropolitan area.

## Statistics analysis

Stata software version 18 and SPSS 22.0 were used to perform statistical analyses. First, the baseline demographic characteristics of the patients were summarized with descriptive statistics. The normality of the sample data was performed with Shapiro-Wilk test. The quantitative data of normal distribution are expressed by means ± standard deviation, while the categorical variables are expressed by frequency and percentage. The time of OS was calculated from the date of diagnosis to the last follow-up or death from any cause, and CSS was to the last follow-up or death from the cause of cancer (AML). OS and CSS were estimated using the Kaplan-Meier method and COX proportional hazards regression model. Survival comparisons among groups were analyzed with the log-rank test. In all statistical tests, *p*-values < 0.05 were considered statistically significant. Bonferroni correction was used to multiple testing (α = 0.05). GraphPad Prism 8.0.2 was used as the drawing software.

## Results

### The characteristics of the AML patients with t(3;3)/inv(3)

**Fig. 1:**
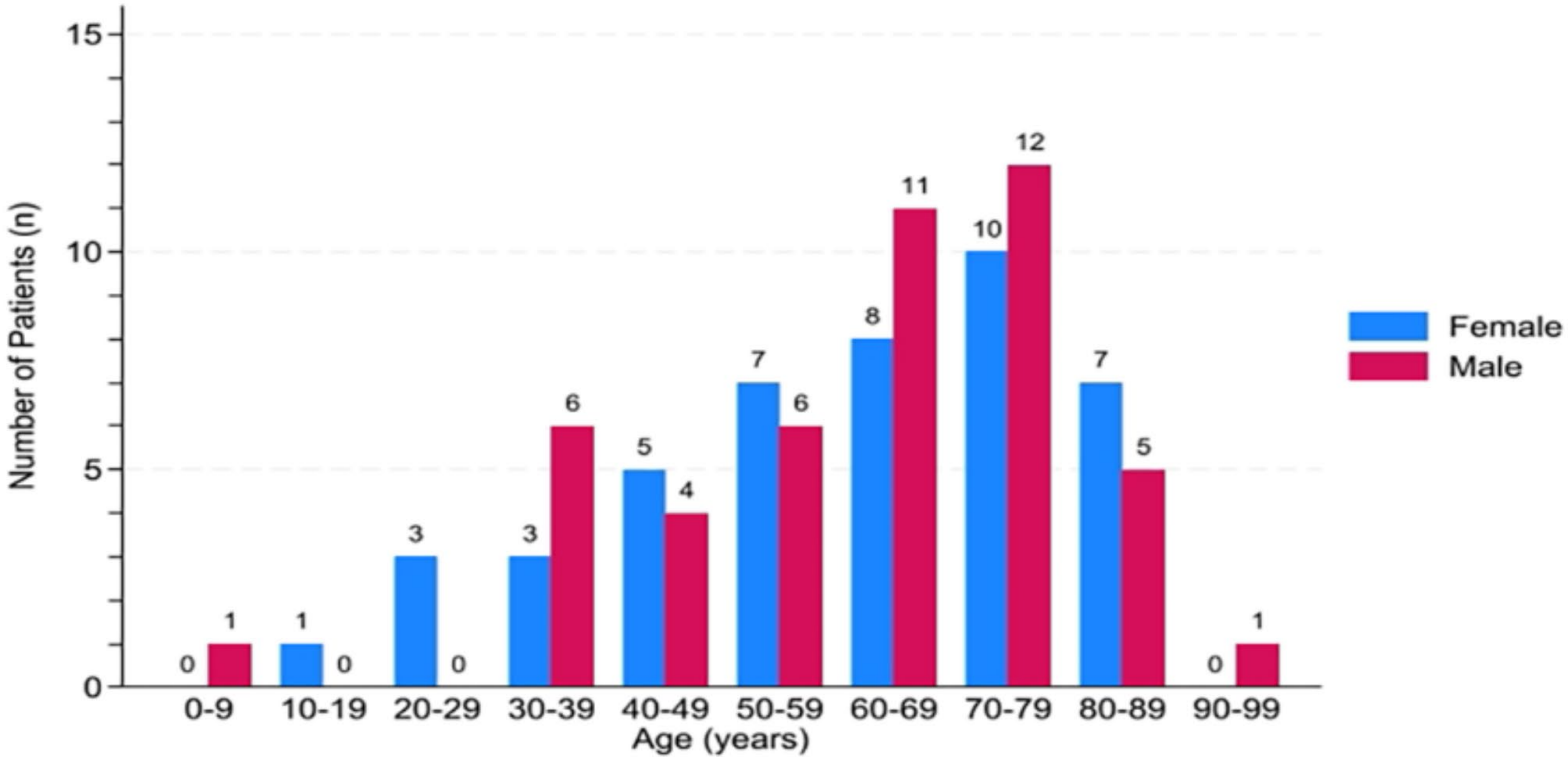
Age distribution based on gender for AML patients with t(3;3)/inv(3) (*n* = 90). The age distribution state of female and male AML patients with t(3;3)/inv(3).

Because of the retrospective study, the missing information on treatments and recurrence, and the limited amount of baseline characteristics available, the provided dataset is not exactly representative for the US population. A total of 90 AML patients with t(3;3)/inv(3) diagnosed between 2010 and 2021 were included in the study. Among them, 44 (48.89%) were female and 46 (51.11%) were male. The median age was 65(interquartile range: 49–74)years. Only two patients were under the age of 18, and they are 9 and 17 years old respectively. There were 54 (60.00%) patients over 60 years old, and 29 (53.70%) were male. The age distribution based on gender is shown in Fig. 1.

The number of patients diagnosed after 2016 was 50 (55.66%). There were 52 (57.78%) patients married, and 16 (17.78%) were never married and single at the time of diagnosis. Of all the patients, 13 (14.44%) were Hispanic (all races), and 77 (85.56%) were non-Hispanic. Among non-Hispanic patients, Whites accounted for 67 (87.01%), the highest proportion. The annual median household income of more than $75,000 patients accounted for 61.11% (55) of the total. Most lived in metropolitan areas (57, 86.66%), especially those with a population of one million people (47, 52.22%). Of these patients, 34 (37.78%) were from the purchased/referred care delivery areas (PRCDA 2020). Of the patients, 76 (84.44%) received chemotherapy, and 14 (15.56%) did not receive chemotherapy or were unknown. The baseline characteristics of the AML patients with t(3;3)/inv(3) are displayed in Table 1.

**Table 1:** Baseline characteristics of AML patients with t(3;3)/inv(3) (*n* = 90).

Baseline Characteristic	Number of patients(*n*/%)
Age at diagnosis (year), median (interquartile range)	65 (49–74)
Age <60 years	36 (40.00%)
Age ≥ 60 years	54 (60.00%)
Sex	
Female	44 (48.89%)
Male	46 (51.11%)
Year of diagnosis	
2010–2015	40 (44.44%)
2016–2021	50 (55.66%)
Marital status at diagnosis	
Married (including common law)	52 (57.78%)
Single (never married)	16 (17.78%)
Divorced	14 (15.56%)
Widowed	7 (7.78%)
Unknown	1 (1.11%)
Race and Ethnicity	
Hispanic (all races)	13 (14.44%)
Non-Hispanic White	67 (74.44%)
Non-Hispanic Black	3 (3.33%)
Non-Hispanic Asian or Pacific Islander	6 (6.67%)
Non-Hispanic American Indian/Alaska Native	1 (1.11%)
Median household income	
<$75,000	35 (38.89%)
≥$75,000	55 (61.11%)
Chemotherapy	
Yes	76 (84.44%)
No/Unknown	14 (15.56%)
PRCDA	
PRCDA	34 (37.78%)
Not PRCDA	56 (62.22%)
Rural-Urban Continuum Code	
Counties in metropolitan areas ge 1 million pop	47 (52.22%)
Counties in metropolitan areas of 250,000 to 1 million pop	22 (24.44%)
Counties in metropolitan areas of lt 250 thousand pop	9 (10.00%)
Nonmetropolitan counties adjacent to a metropolitan area	9 (10.00%)
Nonmetropolitan counties not adjacent to a metropolitan area	3 (3.33%)

The Baseline characteristics of AML patients with t(3;3)/inv(3) include age at diagnosis, sex, year of diagnosis, marital status at diagnosis, race and ethnicity, median household income, chemotherapy, PRCDA, and rural-urban continuum code.

## Survival

### The OS and CSS of total patients

**Fig. 2:**
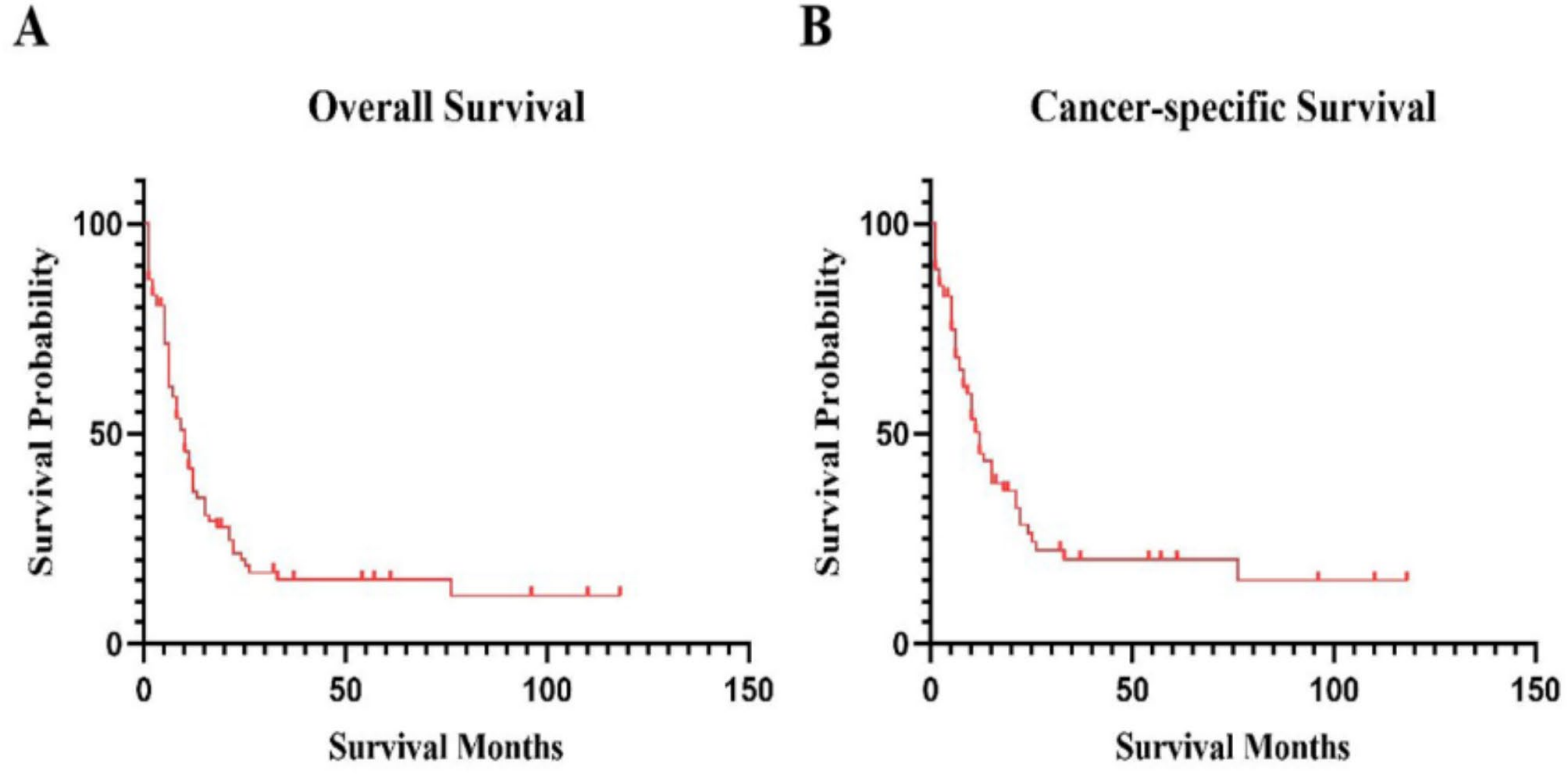
The OS and CSS of total AML patients with t(3;3) /inv(3) (*n* = 90). **A**. The OS of all patients.** B**. The CSS of all patients.

The median OS of the total patients was 8 (3–21) months, and the median CSS was 10 (5–24) months (Fig. 2). The 5-year survival rate for OS was 15.29% (95%CI:7.98-24.78%), and 20.05% (95%CI:10.69–31.51%) for CSS (Tables 3 and 4). The median survival and 5-year survival rate of patients with CSS were superior to those of patients with OS.

### The OS and CSS of patients in different age groups

**Fig. 3:**
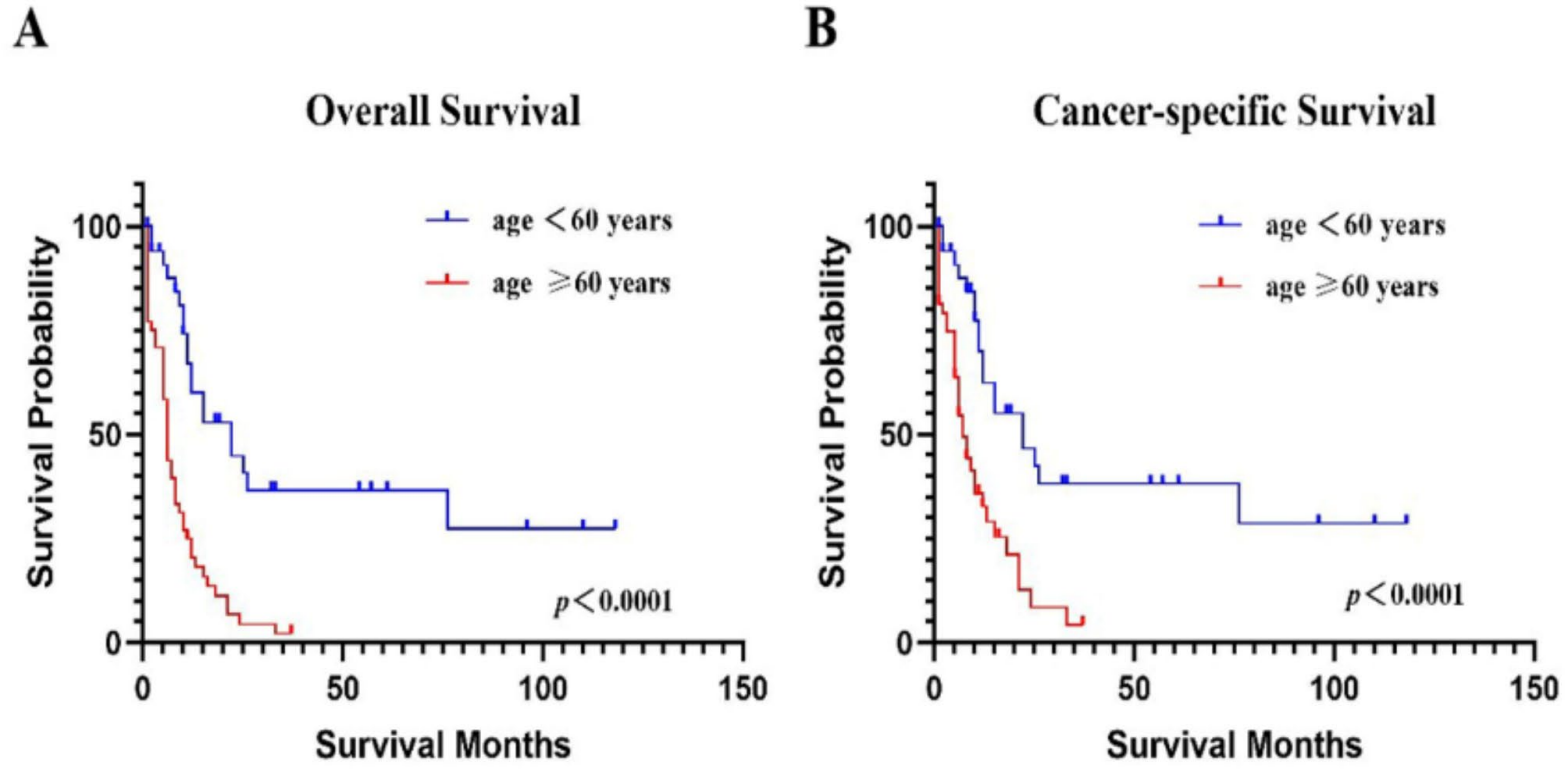
The OS and CSS based on age. **A**. The OS based on age.** B** The CSS based on age.

We divided the patients into age groups of < 60 years and ≥ 60 years. The median OS of < 60 years age group was longer than that in the ≥ 60 age group (15 months vs. 6 months, *p* < 0.001). The median CSS of patients < 60 years was likewise longer than patients ≥ 60 years (22 months vs. 6 months, *p* < 0.001). The 5-year OS and CSS survival rates were 36.65% (19.23-54.28%) and 38.18% (20.09-56.1%) respectively, for patients age<60 years. None of the patients age over 60 years old had a survival time of 5 years (Fig. 3; Table 3, and Table 4).

### The OS and CSS of patients in different sex groups

**Fig. 4:**
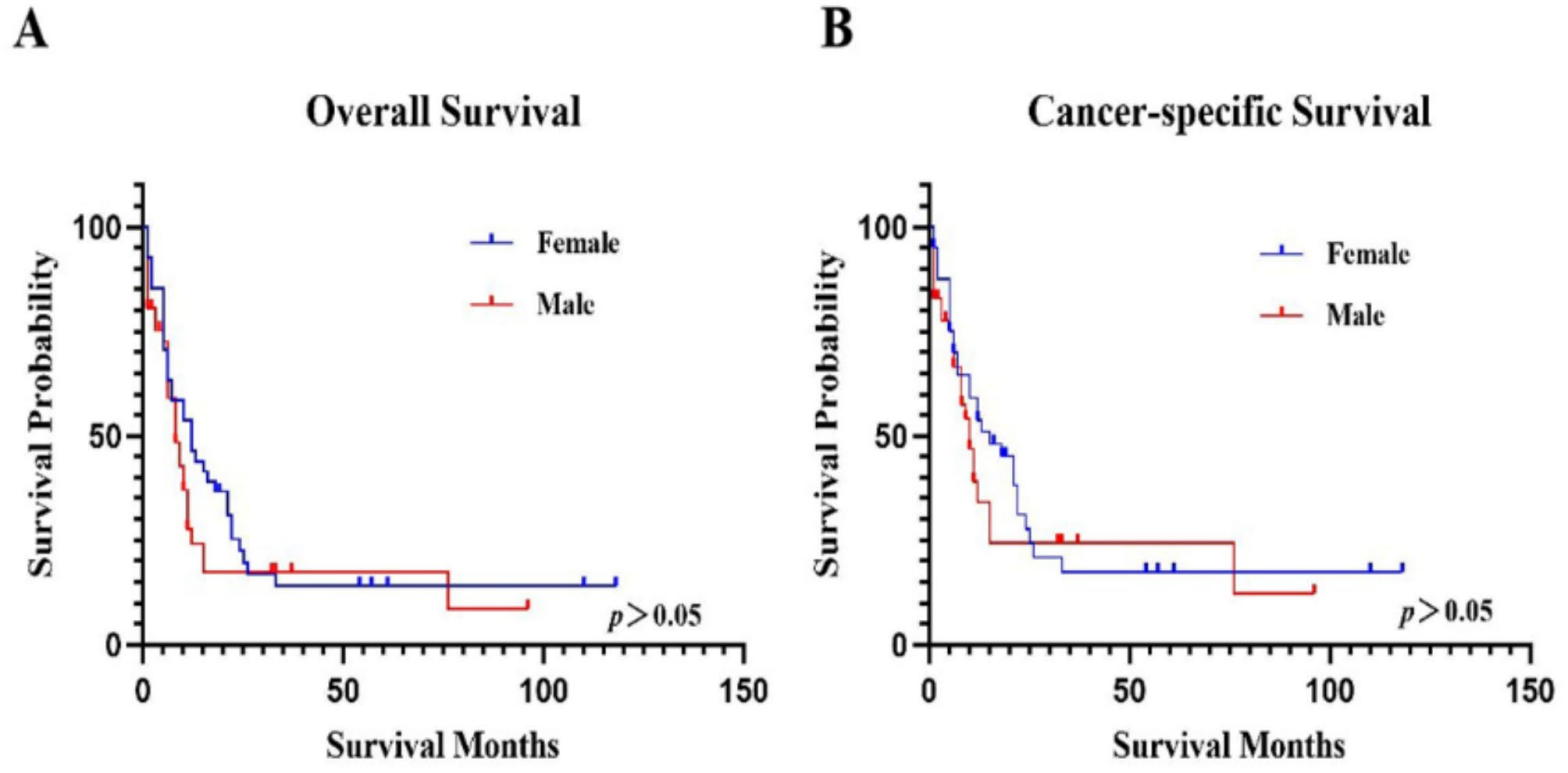
The OS and CSS based on sex. **A**. The OS based on sex.** B**. The CSS based on sex.

The median OS and CSS for female patients were 10 (5–22) months and 13 (5–25) months, while those for males were 8 (1–11) months and 9 (1–15) months, respectively. The 5-year survival rates of OS and CSS for female patients were 14.07% (5.26-27.09%) and 17.35% (6.56-32.43%), and male patients were 17.35% (6.74-32.06%) and 24.40% (9.93-42.25%), respectively. There was no significant difference between male and female patients (*p*>0.05). See Fig. 4; Tables 3 and 4 for further details.

### The OS and CSS of patients in different year of diagnosis

**Fig. 5:**
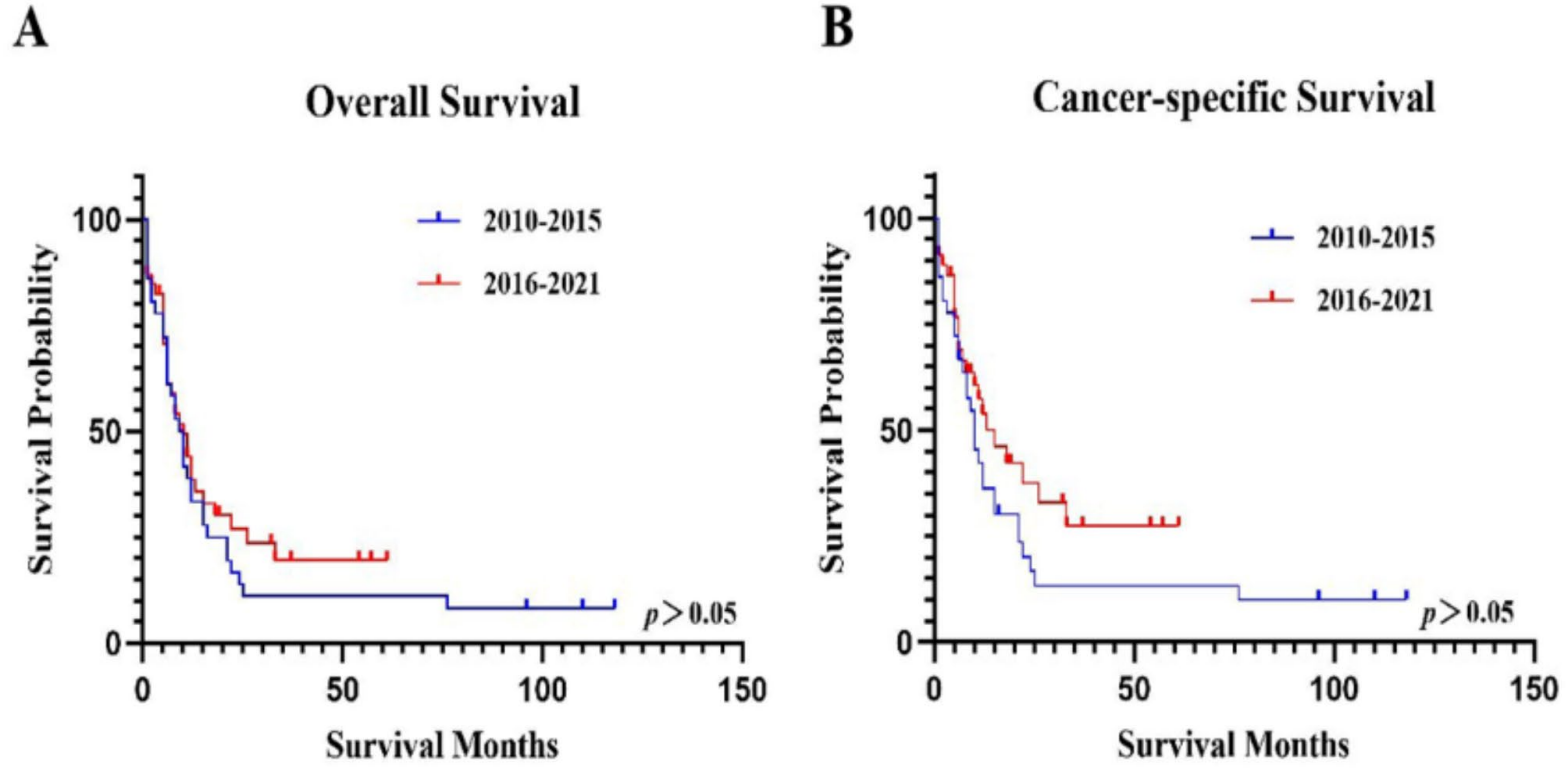
The OS and CSS based on year of diagnosis. **A**. The OS based on year of diagnosis.** B**. The CSS based on year of diagnosis.

All AML patients were diagnosed between 2010 and 2021; therefore, we divided the patients into groups diagnosed in 2010–2015 and 2016–2021 by the year of diagnosis to analyze OS and CSS (Fig. 5; Tables 3 and 4). The median OS of patients diagnosed in 2010–2015 and 2016–2021 was 8 (2–15) months and 8 (5–22) months (*p*>0.05), and the median CSS was 9 (2–21) months and 12 (5-#) months (*p*>0.05). The 5-year survival rate of OS was 11.11% (3.52-23.62%) and 19.64% (8.32-34.45%) for patients diagnosed in 2010–2015 and 2016–2021 (*p*>0.05), and CSS was 13.47% (4.38-27.68%) and 27.44% (11.90-25.58%) (*p*>0.05).

### The OS and CSS of patients in different marital status at diagnosis

**Fig. 6:**
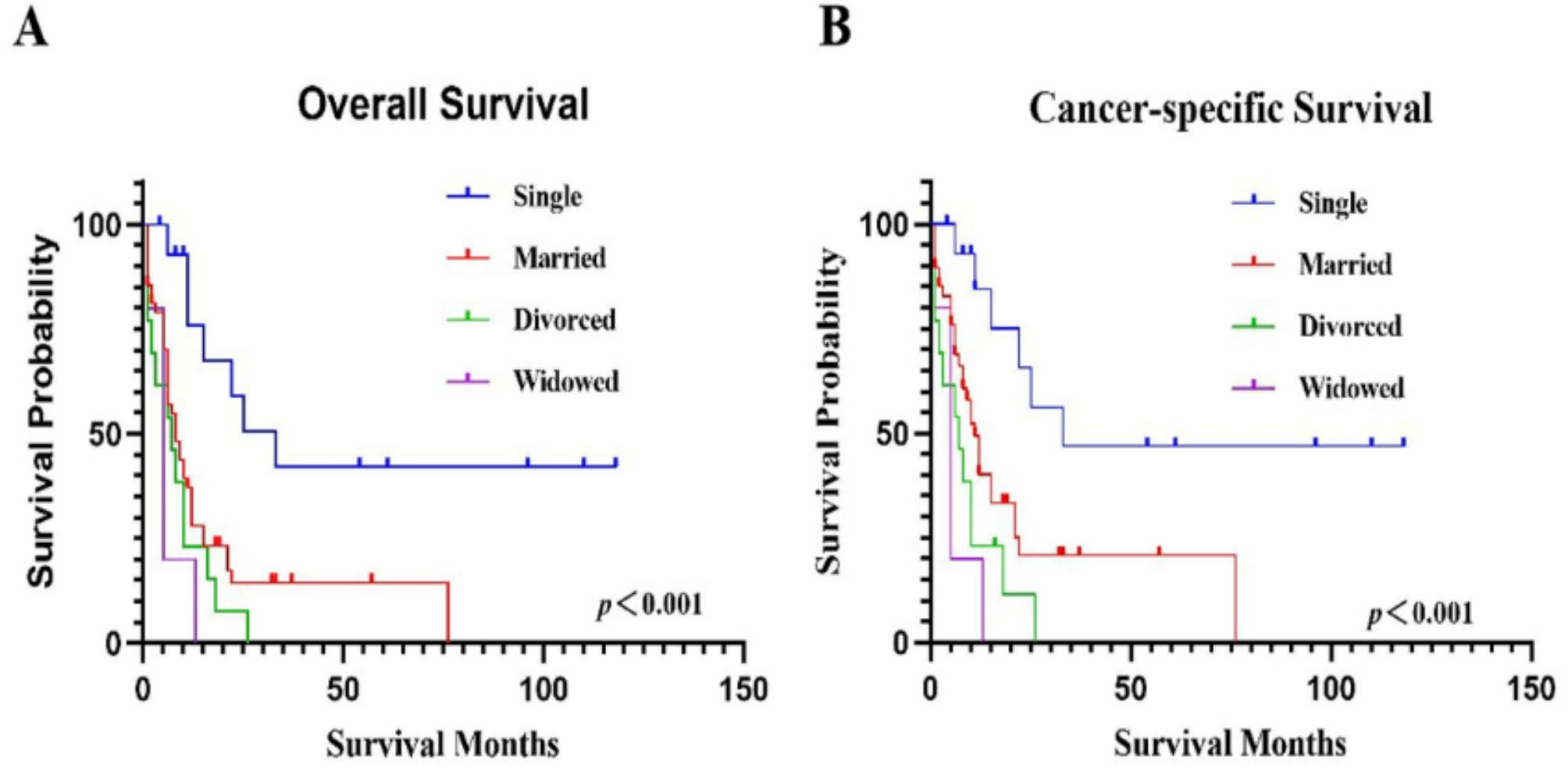
The OS and CSS based on marital status. **A**. The OS based on marital status.** B**. The CSS based on marital status.

We analyzed the effect of marital status on survival and excluded one patient with unknown marital status; therefore, 89 patients were included in the analysis. The median OS of single, married, divorced, and widowed patients were 25 (11-#), 8 (2–15), 6 (1–10), and 5 (0–5) months, respectively. The median CSS were 33 (15-#), 10 (5–21), 6 (1–10), and 5 (0–5) months, respectively. There were significant difference of OS and CSS among patients in different marital status at diagnosis (*p*<0.05). The specific survival curves are shown in Fig. 6. We then performed pairwise comparisons with Bonferroni correction, and adjusted *p*-values<0.008 were considered statistically significant (Table 2). The results showed that the overall survival rate of the single group was higher than that of the married, divorced and widowed groups, and there was no significant difference among the married, divorced and widowed groups. The cancer-specific survival rate of the single group was higher than that of the divorced and widowed groups, and the rate of married group was higher than that of the widowed group. It was further shown that patients who were divorced and widowed had worse survival. The 5-year survival rates of OS and CSS for single patients were 42.21% (15.55-67.05%) and 46.90% (17.53-71.94%), and married patients were 14.56% (5.76-27.24%) and 20.89% (8.47-37.05%), respectively. None of the divorced or widowed patients lived over 5 years (Tables 3 and 4).

**Table 2:** **Comparison of survival rates between different marital status at diagnosis**.

	*p*-value							
Marital status	Single		Married		Divorced		Widowed	
	OS	CSS	OS	CSS	OS	CSS	OS	CSS
Single			0.002	0.009	<0.001	0.001	<0.001	<0.001
Married	0.002	0.009			0.262	0.118	0.034	0.007
Divorced	<0.001	0.001	0.262	0.118			0.235	0.235
Widowed	<0.001	<0.001	0.034	0.007	0.235	0.235		

Bonferroni corrected *p*-values<0.008 were considered statistically significant.

### The OS and CSS based on whether patients received chemotherapy

**Fig. 7:**
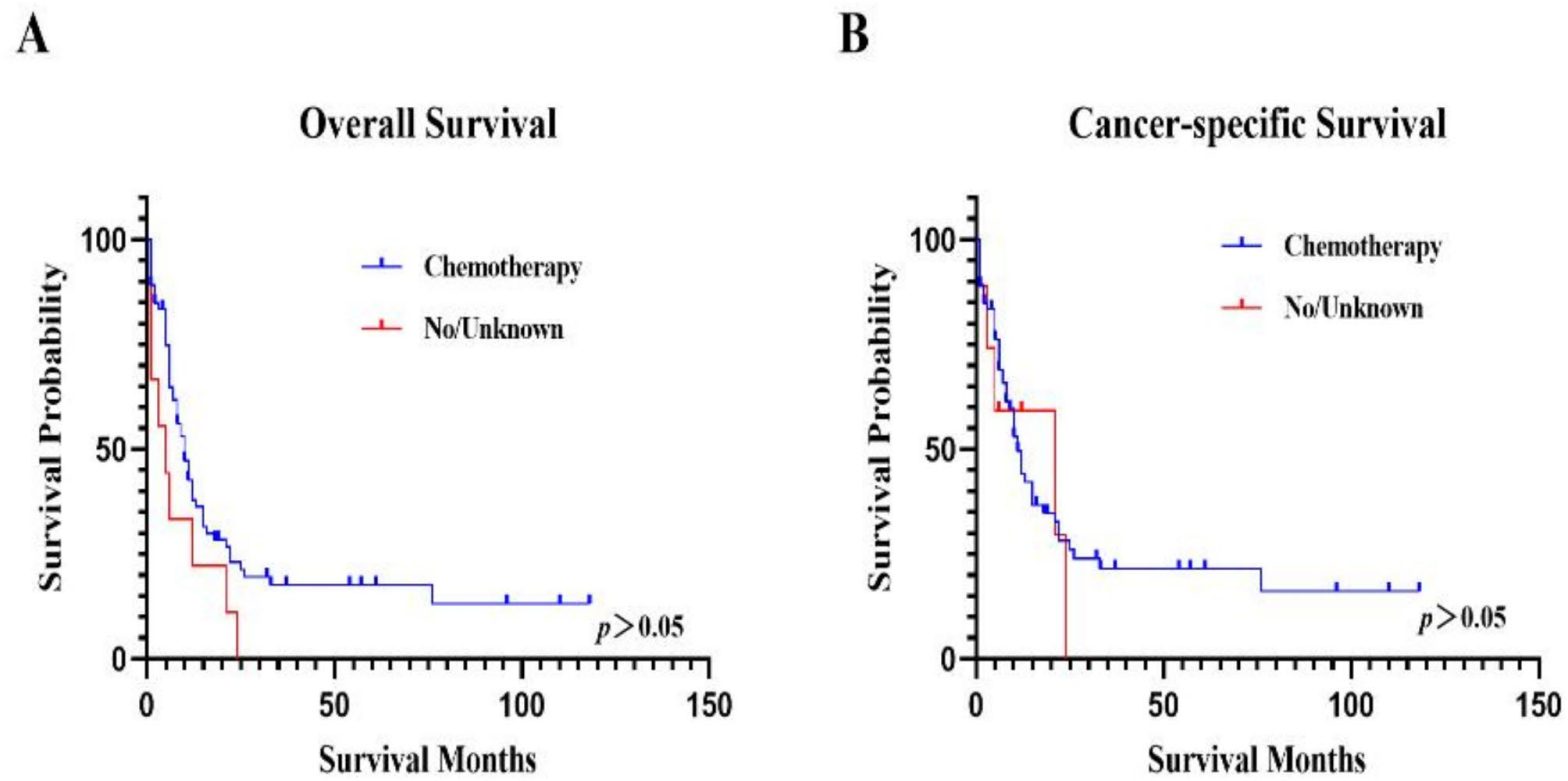
The OS and CSS based on chemotherapy. **A**. The OS based on chemotherapy.** B** The CSS based on chemotherapy. “Yes” representative being received chemotherapy and “No/Unknown” representative not or unknown received chemotherapy.

The median OS of patients with and without chemotherapy were 10 (5–22) months and 1 (0–6) months, and CSS were 11 (5–26) months and 3 (0–21) months, respectively (*p*>0.05). The 5-year survival rates of OS and CSS for patients who received chemotherapy were 17.61% (9.27-28.12%) and 21.54% (11.56-33.53%), respectively. None of the patients who had not received chemotherapy were alive after five years (Fig. 7; Table 3, and Table 4).

Additionally, we also analyzed the OS and CSS based on race and ethnicity, median household income, PRCDA, and rural-urban continuum code. The differences in the median and 5-year survival rates of OS and CSS among the above groups were not significant (*p*>0.05). Detailed data on the median OS, CSS, and 5-year survival rates are presented in Tables 3 and 4.

**Table 3:** Median OS for different demographic groups of AML patients with t(3;3) /inv(3).

Groups	Median OS (in Months)	Median CSS (in Months)
Overall	8 (3–21)	10 (5–24)
Age		
<60 years	15 (10-#)	22 (10-#)
≥60 years	6 (1–10)	6 (1–15)
Sex		
Female	10 (5–22)	13 (5–25)
Male	8 (1–11)	9 (1–15)
Diagnosis year		
2010–2015	8 (2–15)	9 (2–21)
2016–2021	8 (5–22)	12 (5-#)
Marital status at diagnosis		
Married (including common law)	8 (2–15)	10 (5–21)
Single (never married)	25 (11-#)	33 (15-#)
Divorced	6 (1–10)	6 (1–10)
Widowed	5 (0–5)	5 (0–5)
Unknown	*	*
Race and Ethnicity		
Hispanic (all races)	11 (7–13)	11 (7–13)
Non-Hispanic White	8 (2–22)	10 (3–25)
Non-Hispanic Black	6 (1–6)	*
Non-Hispanic Asian or Pacific Islander	5 (3–15)	15 (3-#)
Non-Hispanic American Indian/Alaska Native	8 (8–8)	8 (8–8)
Median household income		
<$75,000	8 (2–15)	9 (2–22)
≥$75,000	8 (3–21)	12 (5–24)
Chemotherapy		
Yes	10 (5–22)	11 (5–26)
No/Unknown	1 (0–6)	3 (0–21)
PRCDA		
PRCDA	10 (5–22)	15 (5–33)
Not PRCDA	7 (2–15)	8 (2–21)
Rural-Urban Continuum Code		
Counties in metropolitan areas ge 1 million pop	8 (2–21)	12 (5–22)
Counties in metropolitan areas of 250,000 to 1 million pop	8 (5–11)	8 (5–12)
Counties in metropolitan areas of lt 250 thousand pop	22 (15-#)	22 (15-#)
Nonmetropolitan counties adjacent to a metropolitan area	6 (5–76)	76 (5–76)
Nonmetropolitan counties not adjacent to a metropolitan area	1 (0–8)	1 (0–8)

* Undefined; Median OS and CSS were not reached in this group at the last follow up date. ^#^ indicates that the survival probability did not reach the corresponding threshold.

**Table 4:** 5-year survival for different demographic groups of AML patients with t(3;3) /inv(3).

Groups	5- Year OS (%, 95%CI)	5-Year CSS (%, 95%CI)
Overall	15.29 (7.98–24.78)	20.05(10.69–31.51)
Age		
<60 years	36.65 (19.23–54.28)	38.18(20.09–56.1)
≥60 years	0	0
Sex		
Female	14.07 (5.26–27.09)	17.35(6.56–32.43)
Male	17.35 (6.74–32.06)	24.40(9.93–42.25)
Diagnosis year		
2010–2015	11.11 (3.52–23.62)	13.47(4.38–27.68)
2016–2021	19.64 (8.32–34.45)	27.44(11.90-25.58)
Marital status at diagnosis		
Married (including common law)	14.56 (5.76–27.24)	20.89(8.47–37.05)
Single (never married)	42.21 (15.55–67.05)	46.90(17.53–71.94)
Divorced	0	0
Widowed	0	0
Unknown	0	0
Race and Ethnicity		
Hispanic (all races)	22.22 (3.37–51.31)	22.22(3.37–51.31)
Non-Hispanic White	16.93 (8.42–27.96)	21.11(10.69–33.90)
Non-Hispanic Black	0	0
Non-Hispanic Asian or Pacific Islander	0	0
Non-Hispanic American Indian/Alaska Native	0	0
Median household income		
<$75,000	17.36 (6.09–33.43)	20.16(7.23–37.66)
≥$75,000	13.98 (5.55–26.20)	19.86(8.09–35.39)
Chemotherapy		
Yes	17.61 (9.27–28.12)	21.54(11.56–33.53)
No/Unknown	0	0
PRCDA		
PRCDA	17.45 (6.13–33.58)	20.82(7.32–38.99)
Not PRCDA	13.77 (5.41–25.98)	20.00(8.27–35.37)
Rural-Urban Continuum Code		
Counties in metropolitan areas ge 1 million pop	13.70 (5.07–26.59)	20.97(8.03–38.01)
Counties in metropolitan areas of 250,000 to 1 million pop	5.00 (0.35–20.53)	6.00(0.42–23.60)
Counties in metropolitan areas of lt 250 thousand pop	0	0
Nonmetropolitan counties adjacent to a metropolitan area	43.75 (10.14–74.19)	58.33(18.02–84.41)
Nonmetropolitan counties not adjacent to a metropolitan area	0	0

“0” represents that no patients are alive.

### COX regression analysis for OS and CSS

Based on the above data collected from the SEER database, we conducted univariate and multivariate COX regression analyses for OS and CSS. We included all categorical variables in Table 1 for a univariate COX regression analysis, and then screened statistically significant categorical variables for a multivariate COX regression analysis. The results of univariate COX regression analysis showed that age at diagnosis, marital status at diagnosis, and chemotherapy significantly affected the prognosis of AML with t(3;3)/inv(3). These variables were then further included in a multivariate COX regression analysis. We found that age and marital status at diagnosis were independent influencing factors for OS and CSS in AML with t(3;3)/inv(3). Compared with age<60 years, age ≥ 60 years increased the risk of overall mortality (HR = 2.407, 95%CI:1.332–4.352) and cancer-specific mortality (HR = 2.407, 95%CI:1.332–4.352). Being divorced and widowed had a high risk of overall and cancer-specific mortality compared with being single (never married). See Tables 5 and 6 for details.

**Table 5:** Univariate and multivariate COX regression analysis for OS.

Variables	Univariate Analysis		Multivariate Analysis	
	HR(95%CI)	*p*-value	HR(95%CI)	*p*-value
Age at diagnosis				
<60 years	Ref.	-	Ref.	-
≥60 years	3.51(2.049–6.014)	<0.001	2.407(1.332–4.352)	0.004
Sex				
Male	Ref.	-	-	-
Female	0.761(0.476–1.216)	0.253	-	-
Year of diagnosis				
2010–2015	Ref.	-	-	-
2016–2021	0.799(0.501–1.272)	0.344	-	-
Marital status at diagnosis				
Single (never married)	Ref.	-	Ref	-
Married (including common law)	3.076(1.423–6.648)	0.004	2.211(0.989–4.94)	0.053
Divorced	4.36(1.768–10.754)	0.001	3.088(1.2-7.949)	0.019
Widowed	7.038(2.535–19.541)	<0.001	3.551(1.204–10.474)	0.022
Race and Ethnicity				
Hispanic (all races)	Ref.	-	-	-
Non-Hispanic White	0.957(0.485–1.886)	0.898	-	-
Non-Hispanic Black	1.887(0.405–8.788)	0.419	-	-
Non-Hispanic Asian or Pacific Islander	1.336(0.454–3.935)	0.599	-	-
Non-Hispanic American Indian/Alaska Native	1.467(0.186–11.555)	0.716	-	-
Median household income				
<$75,000	Ref.	-	-	-
≥$75,000	0.946(0.59–1.516)	0.817	-	-
Chemotherapy				
No/Unknown	Ref.	-	Ref.	-
Yes	0.321(0.173–0.594)	<0.001	0.521(0.269–1.008)	0.053
PRCDA				
Not PRCDA	Ref.	-	-	-
PRCDA	0.69(0.425–1.121)	0.134	-	-
Rural-Urban Continuum Code				
Counties in metropolitan areas ge 1 million pop	Ref.	-	-	-
Counties in metropolitan areas of 250,000 to 1 million pop	1.312(0.76–2.264)	0.329	-	-
Counties in metropolitan areas of lt 250 thousand pop	0.498(0.195–1.27)	0.144	-	-
Nonmetropolitan counties adjacent to a metropolitan area	0.761(0.32–1.811)	0.537	-	-
Nonmetropolitan counties not adjacent to a metropolitan area	3.123(0.946–10.312)	0.062	-	-

HR: hazard ratio, CI: confidence interval, Ref: reference.

**Table 6:** Univariate and multivariate COX regression analysis for CSS.

Variables	Univariate Analysis		Multivariate Analysis	
	HR(95%CI)	*p*-value	HR(95%CI)	*p*-value
Age at diagnosis				
<60 years	Ref.	-	Ref.	-
≥60 years	2.964(1.677–5.238)	<0.001	2.008(1.07–3.767)	0.003
Sex				
Male	Ref.	-	-	-
Female	0.79(0.474–1.314)	0.363	-	-
Year of diagnosis				
2010–2015	Ref.	-	-	-
2016–2021	0.677(0.407–1.125)	0.132	-	-
Marital status at diagnosis				
Single (never married)	Ref.	-	Ref	-
Married (including common law)	2.879(1.251–6.625)	0.013	2.285(0.964–5.416)	0.061
Divorced	4.805(1.838–12.562)	0.001	3.688(1.352–10.061)	0.011
Widowed	8.268(2.843–24.043)	<0.001	4.874(1.566–15.169)	0.006
Race and Ethnicity				
Hispanic (all races)	Ref.	-	-	-
Non-Hispanic White	0.819(0.412–1.628)	0.568	-	-
Non-Hispanic Black	0(0–0)	0.977	-	-
Non-Hispanic Asian or Pacific Islander	0.78(0.213–2.854)	0.708	-	-
Non-Hispanic American Indian/Alaska Native	1.46(0.185–11.508)	0.719	-	-
Median household income				
<$75,000	Ref.	-	-	-
≥$75,000	0.807(0.487–1.338)	0.406	-	-
Chemotherapy				
No/Unknown	Ref.	-	Ref.	-
Yes	0.418(0.202–0.862)	0.018	0.64(0.296–1.384)	0.257
PRCDA				
Not PRCDA	Ref.	-	-	-
PRCDA	0.688(0.406–1.166)	0.165	-	-
Rural-Urban Continuum Code				
Counties in metropolitan areas ge 1 million pop	Ref.	-	-	-
Counties in metropolitan areas of 250,000 to 1 million pop	1.635(0.913–2.929)	0.098	-	-
Counties in metropolitan areas of lt 250 thousand pop	0.662(0.255–1.721)	0.398	-	-
Nonmetropolitan counties adjacent to a metropolitan area	0.8(0.308–2.081)	0.648	-	-
Nonmetropolitan counties not adjacent to a metropolitan area	4.075(1.212–13.698)	0.023	-	-

HR: hazard ratio, CI: confidence interval, Ref: reference.

## Discussion

Acute myeloid leukemia (AML) with t(3;3)/inv(3) is a rare cytogenetic abnormality subtype of AML with adverse outcomes, according to ELN 2022 and WHO 2016^19,27^. The abnormality of t(3;3)/inv(3) results in the aberrant overexpression of the oncogenic transcription factor *EVI1*, promoting proliferation and impairing differentiation of myeloid cells^28–30^. The clinical characteristics and factors affecting the prognosis of AML with t(3;3)/inv(3) have not yet been fully explored as so far. In this study, the data analysis based on population in the United States from the SEER database from 17 registries between 2000 and 2021 was conducted, paying attention to the demographic characteristics and survival outcomes of AML patients with t(3;3)/inv(3). The SEER database presently covers approximately 48% of the U.S. population, containing a total of 22 cancer registries in geographic areas^26^. Although this database also has some shortcomings, it can still reflect the demographics of the entire U.S. population to some extent, providing insight into the incidence and survival rates of various cancers throughout the country.

In the SEER database, the ICD-O-3 code is 9869/3 for acute myeloid leukemia with inv(3)(q21;q26.2) or t(3;3)(q21;q26.2), namely RPN1-EVI1, which is now known as translocation of GATA2 to MECOM^31^. The data of AML with t(3;3)/inv(3) in the SEER database are only available from 2010; therefore, the objects of this study were diagnosed between 2010 and 2021, with 90 patients being included in this study. Previous studies have demonstrated that the disease occurred in relatively younger patients^20,23^. Our study confirms this conclusion. The results showed that the median age was 65 (49–74) years old, which was younger than the median age of all AML patients (68 years)^1^. AML with t(3;3)/inv(3) mainly occurred in adult patients and was rare in children with only one patient less than 14 years old. Furthermore, 60.00% of the patients were over 60 years old, and 53.70% were male. Raya et al.^32^ found that the median age was 50, and 45.71% of 35 AML patients with t(3;3)/inv(3) were female. Richard et al. showed that the median age was 63 years old in untreated patients, 48 years in previously treated patients, and 55 years old in all AML patients with t(3;3)/inv(3), and the percentages of males were 58%, 67%, and 63% in sequence^33^. Accordingly, AML with t(3;3)/inv(3) was more young at diagnosis than other types of AML and was more common in males. More attention should be paid to such patients.

In addition, we analyzed other demographic characteristics of AML patients with t(3;3)/inv(3), including year of diagnosis, marital status at diagnosis, race and ethnicity, median household income, chemotherapy, PRCDA, Rural-Urban Continuum Code. More patients at diagnosis were married, non-Hispanic whites, with high median household income, living in metropolitan areas, as well as treated with chemotherapy. Currently, studies on such demographic characteristics in AML patients with t(3;3)/inv(3) are lacking. Therefore, our study can help us understand the basic demographic information of these patients in the United States, with a certain clinical significance. Other characteristics of this population should be noted by our subsequent researchers.

Previous studies have shown that AML with t(3;3)/inv(3) is rare, with dismal outcomes^20,31,33^. We studied the survival prognosis of AML patients with t(3;3)/inv(3) by analyzing OS and CSS. The median OS and CSS were 8 months and 10 months, and the 5-years OS and CSS probabilities were only 15.29% and 20.05%, indicating poor survival. Similar to our study, Rogers et al.^21^ reported that AML patients with t(3;3)/inv(3) shared a demoralizing outcome with a median OS of less than one year. The median OS was 7.9 (95%CI: 4-12.9) months in newly diagnosed patients and 5.9 (95%CI: 4.4-9) months in relapsed/refractory patients, which were all diagnosed at or referred to the MD Anderson Cancer Center^33^. The 10-years OS probabilities of AML patients with t(3;3)/inv(3) from the United Kingdom Medical Research Council trials was merely 3%^5^. One-year and 4-year OS probabilities were 41% and 13% in Sitges’s research^34^. Fonatsch et al.^35^ found that 15 of 18 AML patients with t(3;3)/inv(3) died within 10 months after diagnosis. According to ELN-2022 stratification system, AML patients with t(3;3)/inv(3) have significantly worse OS than other adverse-risk patients, such as those with t(6;9) (p22.3;q34.1), t(v;11q23.3), and MDS-related genes^36^. Overall, AML patients with t(3;3)/inv(3) have depressing long-term survival, and the prognosis has not been improved over the past few decades. We need to invest more time and resources to explore better treatment options and improve its prognosis. What remains to be done is to identify the risk factors and confounding factors associated with survival of AML patients with t(3;3)/inv(3).

It is urgent to explore the factors affecting the prognosis of AML patients with t(3;3)/inv(3). Therefore, we performed survival analysis in groups based on the above baseline characteristics. The OS and CSS of the patients under 60 years old were both superior to the patients over 60 years old. The 5-years OS and CSS probabilities in patients less than 60 years were 36.65% and 38.18%, respectively. However, none of the patients over the age of 60 survived for 5 years. Weisser’s^20^ study exhibited that the median OS of t(3;3)/inv(3) AML patients with respect to age above and below 60 years was 4 months and 18 months, respectively. Older age was associated with worse OS in the univariate analysis^33^. Therefore, we concluded that elderly patients of AML with t(3;3)/inv(3) have a poorer prognosis. Single (never-married) patients had better OS and CSS than patients with other marital status. We speculated that this may be because single patients were younger. In our study, the median age of single patients was 43 years, which was less than the median age of all patients (65 years), and the median age of married, divorced, and widowed patients were 64, 66, and 84 years, respectively. Age and marital status at diagnosis were independent influencing factors for OS and CSS in AML with t(3;3)/inv(3) through univariate and multivariate COX regression analyses. We can confirm that age and marital status are independent risk factors for AML patients with t(3;3)/inv(3), and others are confounding factors. The identification of such risk factors has a better effect on our subsequent clinical diagnosis and treatment activities. Younger and single patients have better survival, suggesting that we need to pay more attention to survival management in elderly, widowed and divorced patients. For these patients, we can provide better treatment regimen and standardized management process to prolong their survival time.

Currently, our study is the first to investigate the demographic characteristics and survival of AML patients with t(3;3)/inv(3) from the SEER database and is moderately representative of patients in the United States. At present, there are no prognostic studies on the CSS of AML patients with t(3;3)/inv(3). Therefore, our project is somewhat innovative. The SEER database has many advantages, such as large data size, long-term follow-up, multi-dimensional data, high data quality, public availability, coverage of multiple cancers, and policy support. The representativeness and trustworthiness of the SEER database cannot be overlooked, and potential biases may still exist based on differences in access to healthcare and regional differences.

There still remain some limitations of the study to be considered. Firstly, because of the retrospective nature of the study based on SEER database, there may be some selection bias, which affects the accuracy and reliability of the data. Secondly, the SEER database is incomplete and lacks some important clinical information, including blood counts, other cytogenetic information, specific therapeutic regimens, response evaluation. Thirdly, information on follow-up outcomes was limited in SEER database, such as the lack of information on recurrence that precluded the assessment of progression-free survival. Fourthly, underreporting of data and lag in updating the SEER database can affect the accuracy and timeliness of the study. Finally, the small sample size caused low statistical power, so some of the differences observed in our study may not have been detected. To resolve these limitations, we can consider a prospective study design and collect more comprehensive data in the future.

## Conclusion

In conclusion, AML with t(3;3)/inv(3) mostly occurs in adults and has poor clinical outcomes with worse OS and CSS, especially in the elderly. Age and marital status at diagnosis were independent influencing factors for OS and CSS in AML with t(3;3)/inv(3). The prognosis remains depressing with no significant advancements in survival rates over the past few decades. This study highlights the need for a new therapeutic schedule to improve the survival probability of AML patients with t(3;3)/inv(3).

## Electronic supplementary material

Below is the link to the electronic supplementary material.

Supplementary Material 1

## Data Availability

The datasets generated and/or analysed during the current study are available in the SEER (seer.cancer.gov) repository.

## Abbreviations

AML: acute myeloid leukemia
SEER: Surveillance, Epidemiology, and End Results
OS: overall survival
CSS: cancer-specific survival
WHO: the World Health Organization
ELN: European leukemia network
ICD-O-3: the third edition of the International Classification of Diseases for Oncology
PRCDA: the purchased/referred care delivery areas
